# Comprehensive Fractal Description of Porosity of Coal of Different Ranks

**DOI:** 10.1155/2014/490318

**Published:** 2014-05-13

**Authors:** Jiangang Ren, Guocheng Zhang, Zhimin Song, Gaofeng Liu, Bing Li

**Affiliations:** ^1^College of Resources and Environment, Henan Polytechnic University, Jiaozuo, Henan 454003, China; ^2^State Key Laboratory Cultivation Base for Gas Geology and Gas Control, Henan Polytechnic University, Jiaozuo, Henan 454003, China; ^3^College of Resources and Environment, Henan Institute of Engineering, Zhengzhou, Henan 451191, China

## Abstract

We selected, as the objects of our research, lignite from the Beizao Mine, gas coal from the Caiyuan Mine, coking coal from the Xiqu Mine, and anthracite from the Guhanshan Mine. We used the mercury intrusion method and the low-temperature liquid nitrogen adsorption method to analyze the structure and shape of the coal pores and calculated the fractal dimensions of different aperture segments in the coal. The experimental results show that the fractal dimension of the aperture segment of lignite, gas coal, and coking coal with an aperture of greater than or equal to 10 nm, as well as the fractal dimension of the aperture segment of anthracite with an aperture of greater than or equal to 100 nm, can be calculated using the mercury intrusion method; the fractal dimension of the coal pore, with an aperture range between 2.03 nm and 361.14 nm, can be calculated using the liquid nitrogen adsorption method, of which the fractal dimensions bounded by apertures of 10 nm and 100 nm are different. Based on these findings, we defined and calculated the comprehensive fractal dimensions of the coal pores and achieved the unity of fractal dimensions for full apertures of coal pores, thereby facilitating, overall characterization for the heterogeneity of the coal pore structure.

## 1. Introduction

A coal reservoir is a kind of double pore strata, being composed of a matrix pore and a fracture. The aperture structure of coal serves as the basis for research on the occurrence of coal bed methane, the physical and chemical action between the gas-water medium, and the coal matrix block, as well as desorption, diffusion, and seepage of coal bed methane [1–5].

Coal is characterized by the heterogeneity of its surface and structure, mainly embodied in the unevenness of the surface of the coal as well as in the pores of different sizes and shapes in the coal structure. This kind of heterogeneity plays a decisive role in the adsorption process [6]. When conducting research on the pore structure of coal, characterization must be conducted on multiple aspects, including the specific surface area, aperture distribution, and coal heterogeneity. The research shows that the pore distribution and surface morphology of coal have heterogeneity and statistical fractal characteristics; thus, it is difficult to describe them with Euclidean geometry, while the use of fractal geometry is much more suitable for their description [7–11]. When using the fractal dimensions of coal to conduct characterization for its heterogeneity, usually we employ the mercury intrusion method and liquid nitrogen adsorption method to test and calculate the fractal dimensions of the coal [12, 13]. In most cases, the fractal dimensions calculated by these two methods are overlapped in the aperture range between 5.5 nm and 350 nm. Since these two methods are based on different principles, the results obtained are different. Therefore, we must seek a reasonable method to obtain the fractal dimensions for full apertures of coal pores, realize the unity of the data, and calculate the comprehensive fractal dimension.

With the purpose of solving the problems mentioned above, we used the mercury intrusion method and low-temperature liquid nitrogen adsorption method to analyze the pore structure and pore shape of coal; according to the data obtained from the mercury intrusion experiment and low-temperature liquid nitrogen adsorption experiment, we calculated the fractal dimensions of different aperture segments of coal, defined and calculated the comprehensive fractal dimensions of the coal pore, and conducted characterization for coal pore distribution heterogeneity.

## 2. Coal Sample and Experiment

The coal samples used in this experiment included the following: lignite from the Shandong, Longkou Beizao Mine (Number 1); gas coal from the Shandong, Weishan, Caiyuan Mine (Number 2); coking coal from the Shanxi, Gujiao, Xiqu Mine (Number 3); and anthracite from the Henan, Jiaozuo, Guhanshan Mine (Number 4). Sample preparations were in accordance with GB/T 3723-1999. The analytical results for the coal quality are shown in Table 1.

The AutoPore IV 9505 automatic mercury intrusion instrument and the ASAP2020M automatic specific surface analyzer produced by the American Micromeritics Instrument Company were, respectively, used in the mercury intrusion experiment and liquid nitrogen adsorption experiment. The former can test pores from the coal samples with a diameter greater than 5.5 nm, and the latter can test pores with a diameter range between 2 nm and 361 nm. The sizes of coal samples in the mercury intrusion experiment were 3~6 mm. The granularity of coal samples in liquid nitrogen adsorption experiment were 0.17~0.25 mm. The two experiments were carried out at the Engineering Center of the Coal Mine Disaster Prevention and Disaster Relief Education Department at Henan Polytechnic University.

## 3. Experimental Results and Discussion

### 3.1. Experimental Results

Decimal classification of XO*Д*OT (1961) was used [14], and the test results are shown in Tables 2, 3, 4, and 5.

### 3.2. Fractal Dimensions of Coal Pores Calculated Using Mercury Intrusion Method

In accordance with the principle of calculating fractal dimensions of coal pores using the mercury intrusion method [12], we obtained the following formula:

(1) $$\begin{array}{c} \log\left[\frac{{dV}_{p(r)}}{dp\left(r\right)}\right]\propto \left(4-D_{1}\right)\log r\propto \left(D_{1}-4\right)\log p\left(r\right). \end{array}$$

We drew diagrams with log⁡⁡[*dV*_*p*(*r*)_/*dp*(*r*)] and log⁡⁡*p*(*r*) and obtained the slope *K* and then *D*_1_ − 4 = *K*; namely,

(2) $$\begin{array}{c} D_{1}=4+K, \end{array}$$

where *dV*_*p*(*r*)_ is the total pore volume under given pressure (equal to the volume of the mercury injected into the pore); *D*_1_ is the fractal dimension of pore volume (mercury intrusion method); *p*(*r*) is the applied pressure, MPa; and  *r*  is the pore diameter of coal sample, nm.

**Figure 1:**
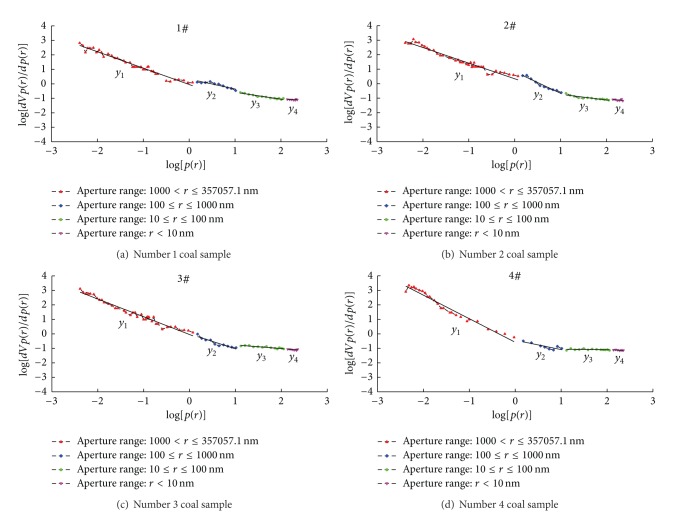
Statistical relationship between  log⁡[*dV*_*p*(*r*)_/*dp*(*r*)]  and  log⁡[*p*(*r*)].

The diagrams of the statistical relationship between log⁡⁡[*dV*_*p*(*r*)_/*dp*(*r*)] and log⁡⁡[*p*(*r*)] of the four coal samples were drawn according to the original data from the mercury intrusion experiment (see Figure 1).

From Figure 1, we can calculate the fractal dimension of coal sample pore distribution using the mercury intrusion method (Table 6).

The results show that, when the aperture of lignite (Number 1), gas coal (Number 2), and coking coal (Number 3) is greater than or equal to 10 nm, the correlation between log⁡⁡[*dV*_*p*(*r*)_/*dp*(*r*)] and log⁡⁡[*p*(*r*)] is significant; the correlation coefficients are all greater than 80%, and the coal pores in the pore segment have obvious fractal characteristics. When the aperture is less than 10 nm, the correlation between log⁡⁡[*dV*_*p*(*r*)_/*dp*(*r*)] and log⁡⁡[*p*(*r*)] is not significant; the correlation coefficients are all smaller than 50%, and the coal pores in the pore segment do not have fractal characteristics. When the aperture of anthracite (Number 4) is greater than or equal to 100 nm, the correlation between log⁡⁡[*dV*_*p*(*r*)_/*dp*(*r*)] and log⁡⁡[*p*(*r*)] is significant; the correlation coefficients are all greater than 74%, and the coal pores in the pore segment have obvious fractal characteristics. When the aperture is smaller than 100 nm, the correlation between log⁡⁡[*dV*_*p*(*r*)_/*dp*(*r*)] and log⁡⁡[*p*(*r*)] is not significant; the correlation coefficients are all smaller than 50%, and the coal pores in the pore segment do not have fractal characteristics.

### 3.3. Fractal Dimension of Coal Pore Calculated Using Liquid Nitrogen Adsorption Method

From the principle of the calculation of fractal dimensions of coal pores using the liquid nitrogen adsorption method [15, 16], we can obtain the following formula:

(3) $$\begin{array}{c} \text{Ln}\left(\frac{V}{V_{m}}\right)=C+\left(D_{2}-3\right)\left[\text{Ln}\left[\text{Ln}\left(\frac{P_{0}}{P}\right)\right]\right], \end{array}$$

where *V* is the total pore volume under given pressure (equal to the adsorption volume); *V*_*m*_ is the adsorption capacity; *D*_2_ is the fractal dimension of pore volume (liquid nitrogen adsorption method); *P* is the adsorption pressure, MPa; *P*_0_ is the maximum adsorption pressure, MPa; and *C* is the constant.

**Figure 2:**
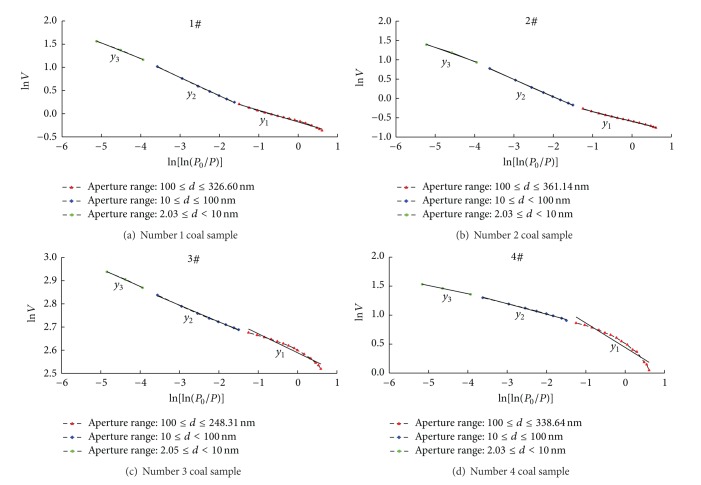
Statistical relationship between Ln *V*  and Ln[Ln(*P*_0_/*P*)].

The diagram of the statistical relationship between Ln *V*  and Ln[Ln(*P*_0_/*P*)] of the four coal samples can be drawn according to the original data from the liquid nitrogen adsorption experiment (see Figure 2).

According to Figure 2, we can calculate the fractal dimension of coal sample pore distribution using the liquid nitrogen adsorption method (Table 7). The results show that, when the aperture range is between 2.03 nm and 361.14 nm, the correlation between Ln *V*  and Ln[Ln(*P*_0_/*P*)] is significant; the correlation coefficients of the four coal samples are all greater than 96%, and the coal pores have obvious fractal characteristics. However, when bounded by apertures of 10 nm and 100 nm, the calculated fractal dimensions are different.

### 3.4. Comprehensive Fractal Dimensions of Coal Pores

Coal is a kind of porous solid, and its surface is inhomogeneous. Quantitative description and characterization can be conducted for the complex structure of a porous solid surface and energy inhomogeneity by introducing fractal dimensions to the research of porous materials. Almost all the solids with highly specific surface area have a fractal dimension between 2 and 3. The closer to 2 the fractal dimension is, the smoother the surface is; while the closer to 3 the fractal dimension is, the rougher the surface is [17–21]. The above-mentioned analyses show that the fractal dimension of coal has important links with its complex pore structure and nonuniform surface area.

Hereby, we used a weighted average in accordance with a specific surface area ratio for the corresponding fractal dimensions of different aperture distribution segments of coal, calling it the “comprehensive fractal dimension of coal,” and denoted it by *D*_*Z*_. Its calculation formula is as follows:

(4) $$\begin{array}{c} D_{Z}=\sum D_{i}\times b_{i}, \end{array}$$

where *D*_*Z*_ is the comprehensive fractal dimension of coal; *D*_*i*_ is the corresponding fractal dimension of *i*th aperture distribution segment; *b*_*i*_ is the specific surface area ratio of the corresponding pore of *i*th aperture distribution segment; and *i* is the *i*th aperture distribution segment, being the positive integer.

The pore parameters of macropores, mesopores, and transition pores in the coal can be measured by the mercury intrusion method, while the pore parameters of partial mesopores, all transition pores, and micropores in the coal can be measured by the liquid nitrogen adsorption method [22, 23]. By comparing the data in Tables 6 and 7, when we calculated the fractal dimensions of transition pores, the precision of the liquid nitrogen adsorption method was higher than that of the mercury intrusion method (the correlation coefficients of the former are all greater than those of the latter). Therefore, when the aperture *d* was greater than 100 nm, we used the mercury intrusion method to calculate the fractal dimension; when the aperture *d* was greater than 2 nm and less than or equal to 100 nm, we used the liquid nitrogen adsorption method to calculate fractal dimension; finally, we used a weighted average for the fractal dimensions obtained so as to arrive at the comprehensive fractal dimension. The results are shown in Table 8.

## 4. Conclusions

1. The results of calculating the fractal dimension of coal using the mercury intrusion method show the following: when the aperture of lignite, gas coal, and coking coal is greater than or equal to 10 nm, the coal pores in the pore segment have obvious fractal characteristics; when the aperture is smaller than 10 nm, the coal pores in the pore segment do not have fractal characteristics; when the aperture of anthracite is greater than or equal to 100 nm, the coal pores in the pore segment have obvious fractal characteristics; and when the aperture is smaller than 100 nm, the coal pores in the pore segment do not have fractal characteristics.
2. The results of calculating the fractal dimension of coal using the liquid nitrogen adsorption method show that, when the aperture range is between 2.03 nm and 361.14 nm, the coal pores have obvious fractal characteristics. However, when bounded by apertures of 10 nm and 100 nm, the calculated fractal dimensions are different.
3. We defined and calculated the comprehensive fractal dimensions of coal pores, introduced them to the research field of the nonuniformity of coal, achieved the effective unity of the fractal dimensions for full apertures of coal pores, and perfected the fractal research of coal pores, thereby facilitating the characterization for the nonuniformity of coal.

## Figures and Tables

**Table 1:** Coal quality analysis for coal sample.

Sample number	*M*_ad_ (%)	*A*_ad_ (%)	*V*_daf_ (%)	*R*_*o*,max⁡_ (%)	Porosity (%)
Number 1	1.69	6.45	36.33	0.38	7.25
Number 2	2.35	8.15	34.57	0.86	5.00
Number 3	0.61	11.14	18.59	1.60	3.42
Number 4	1.26	6.31	7.71	3.49	4.61

Note: the “*M*_ad_” is moisture, the “*A*_ad_” is ash, and the “*V*_daf_” is volatile matter of proximate analysis in coal. The “*R*_*o*,max⁡_” is the maximum reflectance of vitrinite.

**Table 2:** Experimental results of pore volume for coal samples obtained using mercury intrusion method.

Sample number	Pore volume (mL/g)					Pore volume ratio (%)			
	*V*_1_	*V*_2_	*V*_3_	*V*_4_	*V*_*t*_	*V*_1_/*V*_*t*_	*V*_2_/*V*_*t*_	*V*_3_/*V*_*t*_	*V*_4_/*V*_*t*_
Number 1	0.0024	0.0066	0.0121	0.0092	0.0303	7.92	21.78	39.93	30.36
Number 2	0.0067	0.0071	0.0098	0.0086	0.0322	20.81	22.05	30.43	26.71
Number 3	0.0032	0.0027	0.0116	0.0095	0.027	11.85	10.00	42.96	35.19
Number 4	0.0007	0.001	0.0084	0.0086	0.0187	3.74	5.35	44.92	45.99

Note: the subscript “*t*” is total pore, “1” is macropore (Φ > 1000 nm), “2” is mesopore (1000 nm ≧ Φ > 100 nm), “3” is transition pore (100 nm ≧ Φ > 10 nm), and “4” is micropore (10 nm ≧ Φ > 5.5 nm).

**Table 3:** Experimental results of specific surface areas of coal samples obtained by mercury intrusion methods.

Sample number	Specific surface area of pore (m²/g)					Specific surface area ratio of pore (%)			
	*S*_1_	*S*_2_	*S*_3_	*S*_4_	*S*_*t*_	*S*_1_/*S*_*t*_	*S*_2_/*S*_*t*_	*S*_3_/*S*_*t*_	*S*_4_/*S*_*t*_
Number 1	0.004	0.095	2.074	4.986	7.159	0.06	1.33	28.97	69.65
Number 2	0.009	0.081	1.751	4.651	6.493	0.14	1.25	26.97	71.64
Number 3	0.005	0.039	2.103	5.124	7.271	0.07	0.54	28.92	70.47
Number 4	0.001	0.017	1.628	4.631	6.277	0.02	0.27	25.94	73.78

Note: the aperture structure classification is the same as that of Table 2.

**Table 4:** Experimental results of pore volume of coal samples obtained by liquid nitrogen adsorption method.

Sample number	Pore volume (mL/g)				Pore volume ratio (%)		
	*V*_2_	*V*_3_	*V*_4_	*V*_*t*_	*V*_2_/*V*_*t*_	*V*_3_/*V*_*t*_	*V*_4_/*V*_*t*_
Number 1	0.002571	0.003403	0.001164	0.007138	36.02	47.67	16.31
Number 2	0.00243	0.00291	0.000679	0.006019	40.37	48.35	11.28
Number 3	0.002068	0.005161	0.007531	0.01476	14.01	34.97	51.02
Number 4	0.001212	0.002502	0.003773	0.007487	16.19	33.42	50.39

Note: the subscript “4” is the pore volume of micropore (10 nm ≧ Φ > 2 nm) and others are same as Table 2.

**Table 5:** Experimental results of specific surface area of coal samples obtained by liquid nitrogen adsorption method.

Sample number	Specific surface area of pore (m²/g)				Specific surface area ratio of pore (%)		
	*S*_2_	*S*_3_	*S*_4_	*S*_*t*_	*S*_2_/*S*_*t*_	*S*_3_/*S*_*t*_	*S*_4_/*S*_*t*_
Number 1	0.066	0.449	1.377	1.892	3.49	23.73	72.78
Number 2	0.060	0.376	0.71	1.146	5.24	32.81	61.95
Number 3	0.058	0.772	10.457	11.287	0.51	6.84	92.65
Number 4	0.032	0.411	5.169	5.612	0.57	7.32	92.11

Note: the aperture structure classification is the same as that of Table 4.

**Table 6:** Calculation results of fractal dimension of coal sample pore distribution obtained using mercury intrusion method.

Sample number	Aperture range (nm)	Linear fitting equations of different aperture segments	*R* ^2^	*K*	*D*_1_
Number 1	1000 < *r* ≦ 357057.1	*y*_1_ = −1.1308*x* − 0.0460	0.9696	−1.1308	2.8692
	100 ≦ *r* ≦ 1000	*y*_2_ = −0.6854*x* + 0.3292	0.8441	−0.6854	3.3146
	10 ≦ *r* ≦ 100	*y*_3_ = −0.4755*x* − 0.1078	0.9305	−0.4755	3.5245
	*r* < 10	*y*_4_ = −0.0798*x* − 0.9137	0.0688	/	/

Number 2	1000 < *r* ≦ 357057.1	*y*_1_ = −1.0775*x* + 0.3388	0.9593	−1.0775	2.9225
	100 ≦ *r* ≦ 1000	*y*_2_ = −1.4912*x* + 0.8244	0.9601	−1.4912	2.5088
	10 ≦ *r* ≦ 100	*y*_3_ = −0.3893*x* − 0.3467	0.8342	−0.3893	3.6107
	*r* < 10	*y*_4_ = −0.1885*x* − 0.6979	0.1136	/	/

Number 3	1000 < *r* ≦ 357057.1	*y*_1_ = −1.2252*x* − 0.0450	0.9694	−1.2252	2.7748
	100 ≦ *r* ≦ 1000	*y*_2_ = −0.7697*x* − 0.0060	0.8903	−0.7697	3.2303
	10 ≦ *r* ≦ 100	*y*_3_ = −0.2416*x* − 0.5272	0.8091	−0.2416	3.7584
	*r* < 10	*y*_4_ = −0.2049*x* − 0.6250	0.2344	/	/

Number 4	1000 < *r* ≦ 357057.1	*y*_1_ = −1.6239*x* − 0.5805	0.9676	−1.6239	2.3761
	100 ≦ *r* ≦ 1000	*y*_2_ = −0.6457*x* − 0.4407	0.7416	3.3543	3.3543
	10 ≦ *r* ≦ 100	*y*_3_ = −0.0373*x* − 1.0238	0.0863	/	/
	*r* < 10	*y*_4_ = −0.1569*x* − 0.7716	0.3716	/	/

Note: the “*R* ^2^” is coefficient of correlation, the “*K*” is slope, and the “*D*_1_” is fractal dimension of pore volume (mercury intrusion method).

**Table 7:** Calculation results of fractal dimension of coal sample pore distribution obtained by liquid nitrogen adsorption method.

Sample number	Aperture range (nm)	Linear fitting equations of different aperture segments	*R* ^2^	*K*	*D*_2_
Number 1	100 ≦ *d* ≦ 326.60	*y*_1_ = −0.2501*x* − 0.1771	0.9878	−0.2501	2.7499
	10 ≦ *d* < 100	*y*_2_ = −0.3938*x* − 0.3987	0.9994	−0.3938	2.6062
	2.03 ≦ *d* < 10	*y*_3_ = −0.3362*x* − 0.1539	0.9977	−0.3362	2.6638

Number 2	100 ≦ *d* ≦ 361.14	*y*_1_ = −0.2548*x* − 0.5960	0.9969	−0.2548	2.7452
	10 ≦ *d* < 100	*y*_2_ = −0.4473*x* − 0.8511	0.9997	−0.4473	2.5527
	2.03 ≦ *d* < 10	*y*_3_ = −0.3615*x* − 0.4884	0.9963	−0.3615	2.6385

Number 3	100 ≦ *d* ≦ 248.31	*y*_1_ = −0.0822*x* + 2.5891	0.9537	−0.0822	2.9178
	10 ≦ *d* < 100	*y*_2_ = −0.0717*x* + 2.5791	0.9980	−0.0717	2.9283
	2.05 ≦ *d* < 10	*y*_3_ = −0.0757*x* + 2.5721	0.9972	−0.0757	2.9243

Number 4	100 ≦ *d* ≦ 338.64	*y*_1_ = −0.4215*x* + 0.4420	0.9300	−0.4215	2.5785
	10 ≦ *d* < 100	*y*_2_ = −0.1813*x* + 0.6536	0.9956	−0.1813	2.8187
	2.03 ≦ *d* < 10	*y*_3_ = −0.1418*x* + 0.8023	0.9999	−0.1418	2.8582

Note: the “*R* ^2^” is coefficient of correlation, the “*K*” is slope, and the “*D*_2_” is fractal dimension of pore volume (liquid nitrogen adsorption method).

**Table 8:** Calculation results of comprehensive fractal dimension of pore distribution of coal.

Sample number	Aperture range (nm)	*D*_*i*_	Specific surface area (m^2^/g)	Specific surface area ratio (%)	*D*_*z*_
Number 1	*d* > 1000	2.8692	0.004	0.21	2.6552
	100 ≦ *r* ≦ 1000	3.3146	0.095	4.94	
	10 ≦ *r* ≦ 100	2.6638	0.449	23.32	
	2.03 ≦ *d* < 10	2.6062	1.377	71.53	

Number 2	*d* > 1000	2.9225	0.009	0.77	2.5800
	100 ≦ *r* ≦ 1000	2.5088	0.081	6.89	
	10 ≦ *r* ≦ 100	2.6385	0.376	31.97	
	2.03 ≦ *d* < 10	2.5527	0.71	60.37	

Number 3	*d* > 1000	2.7748	0.005	0.04	2.9290
	100 ≦ *r* ≦ 1000	3.2303	0.039	0.35	
	10 ≦ *r* ≦ 100	2.9243	0.772	6.85	
	2.03 ≦ *d* < 10	2.9283	10.457	92.76	

Number 4	*d* > 1000	2.3761	0.001	0.02	2.8231
	100 ≦ *r* ≦ 1000	3.3543	0.017	0.3	
	10 ≦ *r* ≦ 100	2.8582	0.411	7.34	
	2.03 ≦ *d* < 10	2.8187	5.169	92.34	

Note: the “*D*_*i*_” is corresponding fractal dimension of *i*th aperture distribution segment and the “*D*_*z*_” is comprehensive fractal dimension of coal.
